# Objectively measured sedentary time among five ethnic groups in Amsterdam: The HELIUS study

**DOI:** 10.1371/journal.pone.0182077

**Published:** 2017-07-31

**Authors:** Anne Loyen, Mary Nicolaou, Marieke B. Snijder, Ron J. G. Peters, Karien Stronks, Lars J. Langøien, Hidde P. van der Ploeg, Johannes Brug, Jeroen Lakerveld

**Affiliations:** 1 Department of Epidemiology and Biostatistics, Amsterdam Public Health research institute, VU University Medical Center, Amsterdam, the Netherlands; 2 Academic Medical Center, University of Amsterdam, Department: Public Health, Amsterdam Public Health research institute, Amsterdam, the Netherlands; 3 Academic Medical Center, University of Amsterdam, Department: Clinical Epidemiology, Biostatistics and Bioinformatics, Amsterdam Public Health research institute, Amsterdam, the Netherlands; 4 Academic Medical Center, University of Amsterdam, Department: Cardiology, Amsterdam Public Health research institute, Amsterdam, the Netherlands; 5 Department of Physical Education, Norwegian School of Sport Sciences, Olso, Norway; 6 Department of Public and Occupational Health, Amsterdam Public Health research institute, VU University Medical Center, Amsterdam, the Netherlands; 7 Sydney School of Public Health, University of Sydney, Sydney, Australia; 8 Amsterdam School for Communication Research, University of Amsterdam, Amsterdam, the Netherlands; University of Georgia, UNITED STATES

## Abstract

**Introduction:**

Sedentary behaviour is increasingly recognised as a health risk. While differences in this behaviour might help explain ethnic differences in disease profiles, studies on sedentary behaviour in ethnic minorities are scarce. The aim of this study was to compare the levels and the socio-demographic and lifestyle-related correlates of objectively measured sedentary time among five ethnic groups in Amsterdam, the Netherlands.

**Methods:**

Data were collected as part of the HELIUS study. The sample consisted of adults from a Dutch, Moroccan, African Surinamese, South-Asian Surinamese and Turkish ethnic origin. Data were collected by questionnaire, physical examination, and a combined heart rate and accelerometry monitor (Actiheart). Sedentary time was defined as waking time spent on activities of <1.5 metabolic equivalents. Ethnic differences in the levels of sedentary time were tested using ANOVA and ANCOVA analyses, while ethnic differences in the correlates of sedentary time were tested with interactions between ethnicity and potential correlates using general linear models. Associations between these correlates and sedentary time were explored using linear regression analyses stratified by ethnicity (pre-determined). All analyses were adjusted for gender and age.

**Results:**

447 participants were included in the analyses, ranging from 73 to 109 participants per ethnic group. Adjusted levels of sedentary time ranged from 569 minutes/day (9.5 hours/day) for participants with a Moroccan and Turkish origin to 621 minutes/day (10.3 hours/day) in African Surinamese participants. There were no statistically significant differences in the levels or correlates of sedentary time between the ethnic groups. Meeting the physical activity recommendations (150 minutes/week) was consistently inversely associated with sedentary time across all ethnic groups, while age was positively associated with sedentary time in most groups.

**Conclusions:**

No statistically significant differences in the levels of objectively measured sedentary time or its socio-demographic and lifestyle-related correlates were observed among five ethnic groups in Amsterdam, the Netherlands.

## Introduction

Sedentary behaviour, defined as any waking behaviour in a sitting or reclining position and a low energy expenditure, [1] is increasingly recognized as an important health risk. A recent systematic literature review reported positive associations between sedentary time and type 2 diabetes incidence, cancer incidence and mortality, cardiovascular disease incidence and mortality, and all-cause mortality [2]. All-cause mortality has been reported to increase when adults accumulate more than seven to eight hours of sedentary time per day, [3] but engaging in moderate to vigorous physical activity may attenuate, or even eliminate, this risk [2–4]. In 2013, approximately 19 percent of the European adults reported to sit more than 7.5 hours per day [5].

Populations are becoming increasingly ethnically diverse, and the disease risk profiles of ethnic groups differ [6]. For example, the prevalence of cardiovascular risk factors such as type 2 diabetes and hypertension is increased in multiple ethnic minority populations living in the Netherlands [7–9] While these differences between ethnic groups might (partly) be explained by behavioural differences, insight in the levels and underlying correlates of sedentary behaviour across different ethnic groups is currently missing. The aim of the current study was to use data from the Dutch HELIUS study to describe and compare the levels and socio-demographic and lifestyle-related correlates of objectively measured sedentary time among five ethnic groups in Amsterdam.

## Materials and methods

### Study population

The HEalthy LIfe in an Urban Setting (HELIUS) study is a multi-ethnic cohort study conducted in Amsterdam, the Netherlands, aiming to study (the causes of) ethnic differences in cardiovascular and infectious diseases and mental health. Details of this study have been described elsewhere [6]. In brief, baseline data were collected between 2011 and 2015. Potential participants aged 18 to 70 years from a Dutch, Ghanaian, Moroccan, Surinamese and Turkish ethnic origin were randomly sampled (stratified by ethnicity) through the municipality registry from Amsterdam. Data were collected by questionnaire and physical examination. The final sample included nearly 25,000 participants. The HELIUS study was approved by the AMC Ethical Review Board and participants provided written informed consent.

During data collection, HELIUS participants indicated their willingness to participate in future sub-studies. Those individuals that gave permission were invited to participate in the current sub-study, in which detailed information about physical activity and sedentary time was collected, aiming to validate the SQUASH physical activity questionnaire. More information about this sub-study can be found elsewhere [10]. In summary, this study aimed to include 500 participants, with equal distributions between ethnicity and gender. Only Dutch, Moroccan, Surinamese and Turkish participants were invited; Ghanaian participants were excluded from this sub-study because of funding limitations. Data were collected between November 2012 and November 2013 using the Actiheart combined heart rate and accelerometry monitor.

### Variables

#### Ethnicity

Ethnicity was defined according to the country of birth of participants as well as their parents, which is a widely accepted and useful indicator of ethnicity in the Netherlands [11]. Participants were considered to be of non-Dutch origin if they were born abroad and had at least one parent that was born abroad (first generation); or if they were born in the Netherlands but both their parents were born abroad (second generation). For the Dutch sample, people were invited if they and both their parents were born in the Netherlands. Surinamese subgroups (e.g. African, South-Asian or other) were further classified according to self-reported ethnic origin.

#### Sedentary time

Sedentary time was assessed using the Actiheart combined heart rate and accelerometry monitor. The Actiheart (CamNtech Ltd, Papworth, UK) is a small, lightweight and waterproof device that is attached to the chest using two ECG electrodes [12]. Brage and colleagues showed that the Actiheart monitor was a reliable and valid measurement method, especially when the heart rate data and the accelerometry data were combined, [13] and the Actiheart monitor has been used to study sedentary time in previous studies [14]. Participants were invited to the study centre for the calibration and placement of the Actiheart. The Actiheart was individually calibrated using a sub-maximal linear step test following a standardised protocol [13]. Participants who were not physically able to complete the step test, who used medication that might interfere with heart rate measurements (e.g. β-blockers), and pregnant women were excluded. Participants were provided spare ECG stickers and were instructed to wear the Actiheart continuously for five days, including at least one weekend day, and only to remove it when bathing or visiting the sauna.

The Actiheart provided 24-hour data in 15 second epochs. The data were cleaned using the Actiheart software [12]. Participants with missing heart rate data, due to signalling problems, were excluded. Spurious heart rate data (e.g. <30 beats per minute) were either recovered using the inter-beat interval (if possible), interpolated using a straight line (if <5 minutes), or set to missing (if >5 minutes). Subsequently, the data were processed using a branched equation algorithm to indicate time spent at different levels of energy expenditure, defined as metabolic equivalents (METs). Missing energy expenditure data (because of spurious heart rate data >5 minutes or periods of non-wear) were replaced with the average energy expenditure of that day [12]. Therefore, all participants had complete 24-hour data. As sedentary time is defined as *waking* time spent in activities <1.5 MET, and time spent sleeping is usually also characterised by <1.5 MET, sleep time had to be removed from the data. As participants did not keep a diary during the study, a standardised protocol was developed to manually identify sleep time in the Actiheart data. During this process, a researched viewed all of the 24-hour data files and identified the sleep time using a combination of decreased heart rate and reduced acceleration level. Only night time sleeping was considered. This protocol was based on existing protocols [14] and the inter-rater reliability (tested in ten percent of the cases) of the identified sleep time was very high (ICC >0.99). Subsequently, the identified sleep time was extracted from the total time spent at <1.5 MET for each study day, in order to compute daily sedentary time.

#### Potential correlates

Physical activity levels were also assessed by the Actiheart, with time spent at activities >3 METs defined as moderate physical activity and activities >6 METs as vigorous physical activity. Being sufficiently active was defined as accumulating ≥150 minutes per week (operationalised as ≥21.43 minutes per day) of moderate physical activity or ≥75 minutes per week of vigorous physical activity or an equivalent combination [15]. All other variables were derived from the baseline HELIUS data. Age was reported continuously, but since this variable was skewed the median is reported. Educational level was assessed by the highest attained education (combining the education in the Netherlands and in the country of origin) and categorised in no/lower (no, elementary or lower vocational/secondary schooling), intermediate (intermediate vocational or intermediate/higher secondary schooling) and higher (higher vocational schooling or university) education. People were defined as unemployed if they were jobless, incapacitated, or did not belong the labour force (i.e. students, house persons, retirees). Body Mass Index (BMI; kg/m^2^) was calculated from the height and weight recorded during the physical examination and categorised into normal weight (including underweight; BMI <25), overweight (BMI 25–30) and obesity (BMI >30) [16]. Age at migration distinguished between first generation migrants who were ≤12 years, 13–20 years, or >20 years old when they migrated (to distinguish migration during different phases of life [17]) and second generation migrants who were born in the Netherlands. The latter variable was not applicable for participants with a Dutch origin.

### Data handling and statistical analyses

Participants were excluded if they had less than 4 complete days of Actiheart data and/or missing baseline questionnaire data. Differences in mean levels of sedentary time per day between the different ethnic groups were assessed using ANOVA analyses with subsequent post-hoc pairwise analyses with Bonferroni correction, and ANCOVA analyses with gender and age as covariates. To assess differences in correlates of sedentary time between the different ethnic groups, interactions between potential correlates and ethnicity were tested using general linear models, also adjusted for gender and age. For these analyses, statistical significance was defined at p <0.05. The associations between potential correlates and sedentary time in the different ethnic groups were tested using linear regression analyses stratified by ethnicity (pre-determined), again adjusted for gender and age. To account for multiple testing, statistical significance at p <0.05 and p <0.001 (Bonferroni correction) was indicated. All analyses were conducted in SPSS, version 22.

## Results

Of the 462 participants in the HELIUS sub-study, a total of 447 participants provided baseline HELIUS data and had at least four days of complete Actiheart data and were thus included in the current study. The sample characteristics are shown in Table 1. The number of participants per ethnic group ranged from 73 participants (16 percent) with a Moroccan origin to 109 participants (24 percent) with a Dutch origin. The total sample consisted of 53 percent women, ranging from 45 percent in Turkish participants to 61 percent in the Dutch group. The median age of the total sample was 49 years, ranging from 42 years in the Moroccan group to 53 years in the African Surinamese participants. The Dutch participants generally had higher educational levels, were more often employed, and less often obese than the other ethnic groups. Among the first generation migrants, mean age at migration was 16–17 years in participants from a Moroccan and Turkish origin and 21–22 years in both Surinamese groups.

**Table 1:** Sample characteristics of the total sample and the different ethnic groups.

	Total sample	Dutch	Moroccan	African Surinamese	South-Asian Surinamese	Turkish
**N (%) Overall**	447 (100%)	109 (24.4%)	73 (16.3%)	87 (19.5%)	94 (21.0%)	84 (18.8%)
**Gender**						
**N (%) Men**	211 (47.2%)	43 (39.4%)	36 (49.3%)	46 (52.9%)	40 (42.6%)	46 (54.8%)
**N (%) Women**	236 (52.8%)	66 (60.6%)	37 (50.7%)	41 (47.1%)	54 (57.4%)	38 (45.2%)
**Age**						
**Median (IQR) age in years**	49.0 (41.0–54.0)	51.0 (46.5–55.0)	42.0 (34.0–49.0)	53.0 (50.0–56.0)	49.5 (44.8–54.0)	43.0 (36.0–49.0)
**Educational level**						
**N (%) No/lower**	182 (42.1%)	16 (15.0%)	32 (45.7%)	40 (49.4%)	50 (54.3%)	44 (53.7%)
**N (%) Intermediate**	119 (27.5%)	23 (21.5%)	26 (37.1%)	21 (25.9%)	24 (26.1%)	25 (30.5%)
**N (%) Higher**	131 (30.3%)	68 (63.6%)	12 (17.1%)	20 (24.7%)	18 (19.6%)	13 (15.9%)
**Employment status**						
**N (%) Unemployed**	117 (27.1%)	10 (9.3%)	34 (48.6%)	24 (29.3%)	21 (22.8%)	28 (35.0%)
**N (%) Employed**	315 (72.9%)	98 (90.7%)	36 (51.4%)	58 (70.7%)	71 (77.2%)	52 (65.0%)
**Weight status**						
**Median (IQR) BMI in kg/m**^**2**^	26.1 (23.3–29.1)	24.2 (21.7–26.4)	27.3 (23.6–30.3)	26.5 (23.9–31.1)	25.9 (23.7–28.0)	28.0 (25.0–32.0)
**N (%) BMI <25 kg/m**^**2**^	178 (39.8%)	64 (58.7%)	26 (35.6%)	28 (32.2%)	39 (41.5%)	21 (25.0%)
**N (%) BMI 25–30 kg/m**^**2**^	176 (39.4%)	37 (33.9%)	27 (37.0%)	34 (39.1%)	43 (45.7%)	35 (41.7%)
**N (%) BMI >30 kg/m**^**2**^	93 (20.8%)	8 (7.3%)	20 (27.4%)	25 (28.7%)	12 (12.8%)	28 (33.3%)
**Physical activity**						
**Median (IQR) min/day MVPA**	26.9 (15.8–42.4)	30.7 (18.2–43.9)	32.3 (19.2–45.9)	22.3 (11.8–37.6)	20.9 (12.5–33.0)	30.0 (17.0–50.0)
**N (%) Insufficiently active**	165 (36.9%)	32 (29.4%)	21 (28.8%)	41 (47.1%)	44 (46.8%)	27 (32.1%)
N (%) Sufficiently active*	282 (63.1%)	77 (70.6%)	52 (71.2%)	46 (52.9%)	50 (53.2%)	57 (67.9%)
**Age at migration**						
**Mean (SD) age in years**	19.29 (9.39)	N/A	16.57 (10.58)	22.42 (9.27)	20.60 (8.48)	16.18 (8.09)
**N (%) ≤12 years**	53 (15.7%)	N/A	17 (23.3%)	6 (6.9%)	12 (12.8%)	18 (21.4%)
**N (%) 13–20 years**	106 (31.4%)	N/A	17 (23.3%)	33 (37.9%)	27 (28.7%)	29 (34.5%)
**N (%) >20 years**	120 (35.5%)	N/A	20 (27.4%)	39 (44.8%)	42 (44.7%)	19 (22.6%)
**N (%) 2**^**nd**^ **generation**	59 (17.5%)	N/A	19 (26.0%)	9 (10.3%)	13 (13.8%)	18 (21.4%)

BMI Body Mass Index; IQR InterQuartile Range; min minutes; SD Standard Deviation

*Defined as ≥ 150 minutes of moderate to vigorous physical activity per week.

**Fig 1:**
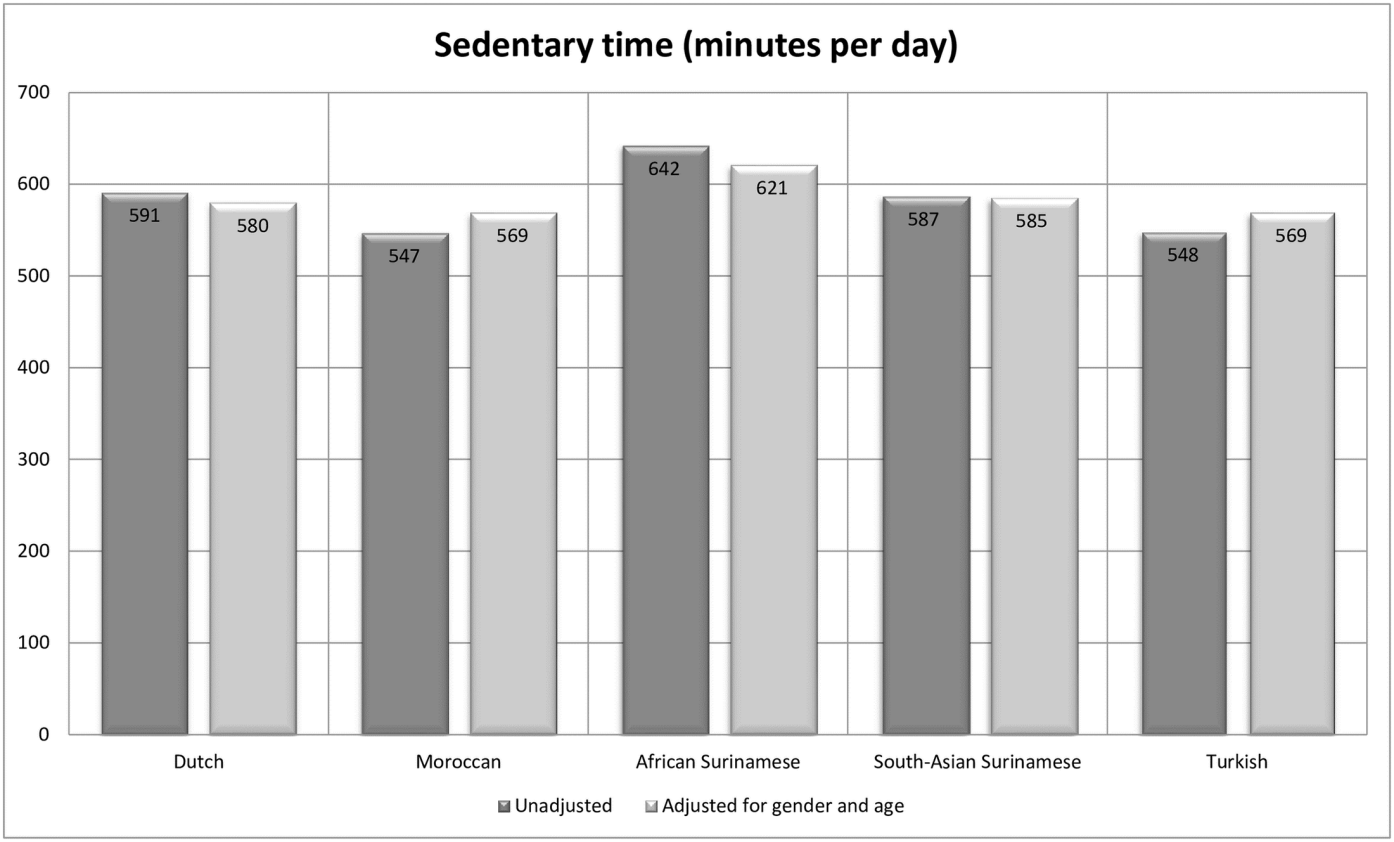
Mean sedentary time (minutes per day) among the different ethnic groups, unadjusted (dark grey) and adjusted for gender and age (light grey).

Overall, participants accumulated a mean of 585 minutes (almost 10 hours) of sedentary time per day. These numbers ranged from 547–548 minutes per day (9.1 hours per day) in Moroccan and Turkish participants, to 642 minutes per day (10.7 hours per day) in participants from an African Surinamese origin. Sedentary time was statistically significantly different across ethnic groups (ANOVA p <0.000), and pairwise post-hoc analyses showed significant differences between the Moroccan and African Surinamese participants, and the Turkish and African Surinamese participants. However, when adjusted for gender and age, the mean minutes per day converged and the differences between the ethnic groups were no longer statistically significant (ANCOVA p <0.158). The unadjusted and adjusted levels of sedentary time per ethnic group are depicted in Fig 1.

The results of the analyses to explore correlates of sedentary time are shown in Table 2. There were no statistically significant interactions between ethnicity and any of the potential correlates in their association with sedentary time (Table 2, last column). Meeting the physical activity recommendations of 150 minutes of moderate to vigorous physical activity per week was consistently associated with lower levels of sedentary time among all ethnic groups. Compared with those not meeting the recommendations, participants meeting the physical activity recommendations accumulated 120–200 minutes less sedentary time per day. Age was positively associated with sedentary time among all ethnic groups except for the African Surinamese, with varying levels of statistical significance. A one-year increase in age seems to be associated with an increase of 4–5 sedentary minutes per day. No clear associations with sedentary time were observed for the other potential correlates.

**Table 2:** Associations of potential correlates and sedentary time (minutes per day) among the different ethnic groups, adjusted for gender and age.

	Dutch	Moroccan	African Surinamese	South-Asian Surinamese	Turkish	Interaction ethnicity
	*B* (95% CI)	*B* (95% CI)	*B* (95% CI)	*B* (95% CI)	*B* (95% CI)	*p*-value
**Gender**						0.297
Men (ref)						
Women	-30.36 (-84.50–23.77)	23.48 (-41.74–88.71)	38.68 (-25.65–103.01)	-36.17 (-95.87–23.53)	-12.44 (-84.41–59.53)	
**Age (continuous)**	***5*.*33 (2*.*39–8*.*26)***	*3*.*77 (0*.*77–6*.*76)*	2.02 (-3.01–7.06)	*4*.*34 (1*.*31–7*.*37)*	*4*.*19 (0*.*41–7*.*98)*	0.775
**Educational level**						0.368
No/low (ref)						
Intermediate	73.11 (-12.28–158.49)	21.66 (-55.22–98.53)	-49.36 (-130.50–31.79)	*81*.*79 (12*.*52–151*.*07)*	30.75 (-53.28–114.77)	
High	54.74 (-19.44–128.91)	19.82 (-77.08–116.71)	42.58 (-38.21–123.36)	-3.04 (-79.68–73.59)	40.23 (-64.95–145.41)	
**Employment status**						0.522
Unemployed (ref)						
Employed	55.26 (-36.53–147.04)	27.40 (-44.49–99.29)	-7.94 (-80.44–64.56)	9.95 (-60.99–80.89)	-50.01 (-127.64–27.61)	
**Weight status**						0.495
BMI <25 kg/m^2^ (ref)						
BMI 25–30 kg/m^2^	9.89 (-45.96–65.75)	4.21 (-73.92–82.34)	3.98 (-73.35–81.32)	49.87 (-14.14–113.88)	-0.10 (-96.50–96.30)	
BMI >30 kg/m^2^	8.97 (-90.79–108.73)	-15.71 (-100.76–69.34)	80.26 (-7.06–167.58)	18.67 (-75.59–112.93)	53.40 (-47.34–154.14)	
**Physical activity**						0.338
Insufficiently active (ref)						
Sufficiently active*	***-158*.*29 (-205*.*74 - -110*.*83)***	***-123*.*51 (-189*.*63 - -57*.*38)***	***-199*.*89 (-248*.*76 - -151*.*03)***	***-170*.*13 (-216*.*89 - -123*.*36)***	***-199*.*37 (-264*.*02 - -134*.*71)***	
**Age at migration**						0.127
≤12 years (ref)	N/A					
13–20 years	N/A	-18.68 (-124.45–87.10)	-74.51 (-215.23–66.21)	20.38 (-84.28–125.04)	*129*.*40 (31*.*33–227*.*46)*	
>20 years	N/A	-32.60 (-157.35–92.15)	-25.08 (-164.36–114.20)	-16.33 (-121.41–88.74)	66.07 (-44.41–176.55)	
2^nd^ generation	N/A	-34.82 (-129.78–60.14)	28.57 (-128.65–185.80)	20.61 (-94.47–135.70)	50.92 (-68.75–170.58)	

Numbers in italic represent p < 0.05.

Numbers in italic and bold represent p < 0.001.

CI Confidence Interval; BMI Body Mass Index

*Defined as ≥ 150 minutes of moderate to vigorous physical activity per week.

## Discussion

The aim of this study was to compare objectively measured levels and correlates of sedentary time between adults with a Dutch, Moroccan, African Surinamese, South-Asian Surinamese and Turkish ethnic origin living in Amsterdam, the Netherlands. Observed levels of sedentary time ranged from around 9 hours per day for participants from a Moroccan and Turkish origin, to more than 10.5 hours per day for African Surinamese participants. However, the differences between ethnic groups were not statistically significant after adjustment for gender and age. In addition, no evidence was found for differences in socio-demographic and lifestyle-related correlates of sedentary time between the ethnic groups. Physical activity and age were the only identified correlates of sedentary time.

Studies comparing sedentary behaviour across ethnic groups are scarce. Therefore, it is difficult to compare these results to previous studies. In addition, the studies that are available generally focus on children and often use self-report measures (e.g. questionnaires) and/or proxy measures (e.g. TV time) to assess sedentary behaviour. Some of these studies did report differences in sedentary time between different ethnic groups or between migrants and the host population [18–22]. For example, van Rossem and colleagues reported that preschool children from a non-Dutch background were more likely to watch television for at least 2 hours per day and to sit in a buggy for at least 0.5 hours per day compared to Dutch children [19]. But because of the differences in study populations and measurement methods, the results of these studies cannot directly be compared to our study.

While this study observed no meaningful differences in total sedentary time across different ethnic groups, earlier studies have shown clear ethnic differences in other cardio-metabolic health-related behaviours, such as dietary [23–25] and physical activity patterns [26]. Specifically, de Munter and colleagues reported that different ethnic groups in the Netherlands tend to be physically active in different domains [26]. This might also be true for sedentary behaviour. As the present study only assessed total sedentary time, further research may explore ethnic differences in sedentary behaviour domains (e.g. sitting during transport, at work, or during leisure time). Especially differences in the time spent TV viewing might be relevant, as TV viewing might be more detrimental for health outcomes than other sedentary behaviours [4, 27].

No evidence was found for differences in socio-demographic and lifestyle-related correlates of sedentary time between the ethnic groups. Physical activity and age were the only potential correlates that were associated with sedentary time.

Across all ethnic groups, meeting the recommendations of 150 minutes of moderate to vigorous physical activity per week was consistently associated with less sedentary time. This association is in line with previous research [28]. Even though sedentary time and physical activity were both measured using the Actiheart, this association is not likely to be merely the effect of time replacement. The relationship between the two behaviours was not inverse as light physical activity (1.5–3.0 METs) was not taken into account, and approximately 20 minutes of moderate to vigorous physical activity per day was associated with 120–200 minutes less sedentary time per day.

Age was associated with higher levels of sedentary time among all ethnic groups except for the African Surinamese, with varying levels of statistical significance. A one-year age difference was associated with 4–5 more sedentary minutes per day. Previous studies (in the general population) have reported mixed results on the associations between age and sedentary time [28].

As previous studies (not focused on ethnic groups) reported gender, educational level and weight status to be associated with sedentary time, [5, 28] it is surprising that these were not identified in the current study, especially in the Dutch participants. Together with the large confidence intervals, this might be an indication that we lacked statistical power in these analyses.

### Strengths and limitations

This is one of the first studies to objectively describe and compare levels and socio-demographic and lifestyle-related correlates of sedentary time among adults of different ethnic groups. Therefore, it provides the first insights in this topic and starting points for further research.

One possible limitation of this study might be the presence of selection bias, as only a selection of the HELIUS participants might have been willing to participate in this particular sub-study. Non-responder analyses indeed showed differences in BMI, gender, and migration generation between responders and non-responders in the sub-study [10]. More specifically, it is possible that more active and less sedentary participants were willing to participate in this sub-study and wear the Actiheart monitor, causing the ethnic groups in the study to be more homogenous than in real life, possibly concealing existing differences in the prevalence of sedentary time between the ethnic groups.

Another possible limitation is the relatively small number of participants per ethnic group (i.e. 73–109 participants) and, consequently, the possible lack of statistical power in the stratified analyses assessing the correlates of sedentary time within those groups. Even though the analyses were adjusted for only a few variables (i.e. gender and age), and only included variables with sufficient numbers per subgroup, the large confidence intervals and the lack of ‘usual’ associations even within the Dutch participants might indicate a lack of statistical power to demonstrate associations between potential correlates and sedentary time in the different groups. Therefore, larger studies focusing on the correlates of sedentary time in ethnic groups are needed.

One of the main strengths of this study is the fact that sedentary time was objectively measured using the validated Actiheart combined heart rate and accelerometry monitor. It is likely that this resulted in more valid and better comparable levels of sedentary time than self-report measures, as self-report measures suffer from recall and social desirability bias and possible linguistic and cultural differences in the interpretation of the questions. The latter might be an especially important consideration in research focussed on differences between cultures or ethnicities, even though Nicolaou and colleagues did not find ethnic differences in the reliability and validity of the SQUASH questionnaire when compared to the Actiheart monitor [10]. Nevertheless, objective measures such as the Actiheart lack contextual and domain specific information, which might have concealed ethnic differences in sedentary time domains. In addition, the Actiheart is not able to assess the posture of participants, making it impossible to distinguish between sitting and standing, while this might have implications for health [29, 30]. Other accelerometers, such as the ActivPAL [31], are able to do so and might thus be preferable in the study of sedentary behaviour. Finally, as participants were not asked to keep a log, a ‘subjective’ method was needed to distinguish sleep time in the 24-hours data. Even though this might have compromised the objective nature of the data, the protocol used to identify sleep time was based on existing protocols, the inter-rater reliability was high, and the mean observed sleep time matched the average sleep time reported by participants.

## Conclusions

This is one of the first studies to assess objectively measured sedentary time across different ethnic groups. No differences in the levels or the socio-demographic and lifestyle-related correlates of objectively measured sedentary time were observed between adults from five ethnic groups in Amsterdam, the Netherlands. Meeting the physical activity recommendations was inversely associated with sedentary time across all ethnic groups while age was positively associated with sedentary time in most groups. Studies with larger subgroups and studies assessing domains of sedentary time are needed to provide more insight in this topic.

## References

[1] Sedentary Behaviour Research Network. Letter to the editor: standardized use of the terms "sedentary" and "sedentary behaviours". Appl Physiol Nutr Metab. 2012;37(3):540–2. doi: 10.1139/h2012-024 22540258 10.1139/h2012-024

[2] Biswas A, Oh PI, Faulkner GE, Bajaj RR, Silver MA, Mitchell MS, et al Sedentary time and its association with risk for disease incidence, mortality, and hospitalization in adults: a systematic review and meta-analysis. Ann Intern Med. 2015;162(2):123–32. doi: 10.7326/M14-1651 25599350 10.7326/M14-1651

[3] Chau JY, Grunseit AC, Chey T, Stamatakis E, Brown WJ, Matthews CE, et al Daily sitting time and all-cause mortality: a meta-analysis. PLoS One. 2013;8(11):e80000 doi: 10.1371/journal.pone.0080000 24236168 10.1371/journal.pone.0080000 PMC3827429

[4] Ekelund U, Steene-Johannessen J, Brown WJ, Fagerland MW, Owen N, Powell KE, et al Does physical activity attenuate, or even eliminate, the detrimental association of sitting time with mortality? A harmonised meta-analysis of data from more than 1 million men and women. The Lancet. 2016;388(10051):1302–10. doi: 10.1016/s0140-6736(16)30370-1 10.1016/S0140-6736(16)30370-1 27475271

[5] Loyen A, van der Ploeg HP, Bauman A, Brug J, Lakerveld J. European Sitting Championship: Prevalence and Correlates of Self-Reported Sitting Time in the 28 European Union Member States. PLoS One. 2016;11(3):e0149320 doi: 10.1371/journal.pone.0149320 26934701 10.1371/journal.pone.0149320 PMC4774909

[6] Stronks K, Snijder MB, Peters RJG, Prins M, Schene AH, Zwinderman AH. Unravelling the impact of ethnicity on health in Europe: the HELIUS study. BMC Public Health. 2013;13:402 doi: 10.1186/1471-2458-13-402 23621920 10.1186/1471-2458-13-402 PMC3646682

[7] Meeks KA, Stronks K, Beune EJ, Adeyemo A, Henneman P, Mannens MM, et al Prevalence of type 2 diabetes and its association with measures of body composition among African residents in the Netherlands-The HELIUS study. Diabetes Res Clin Pract. 2015;110(2):137–46. doi: 10.1016/j.diabres.2015.09.017 26432411 10.1016/j.diabres.2015.09.017

[8] Agyemang C, Kieft S, Snijder MB, Beune EJ, van den Born BJ, Brewster LM, et al Hypertension control in a large multi-ethnic cohort in Amsterdam, The Netherlands: the HELIUS study. Int J Cardiol. 2015;183:180–9. doi: 10.1016/j.ijcard.2015.01.061 25679990 10.1016/j.ijcard.2015.01.061

[9] Snijder MB, Agyemang C, Peters RJ, Stronks K, Ujcic-Voortman JK, Van Valkengoed IGM. Case finding and medical treatment of type 2 diabetes among different ethnic minority groups. The HELIUS study. J Diab Res (in press). 2016. 10.1155/2017/9896849 PMC5244015 28154830

[10] Nicolaou M, Gademan MG, Snijder MB, Engelbert RH, Dijkshoorn H, Terwee CB, et al Validation of the SQUASH Physical Activity Questionnaire in a Multi-Ethnic Population: The HELIUS Study. PLoS One. 2016;11(8):e0161066 doi: 10.1371/journal.pone.0161066 27575490 10.1371/journal.pone.0161066 PMC5004804

[11] Stronks K, Kulu-Glasgow I, Agyemang C. The utility of 'country of birth' for the classification of ethnic groups in health research: the Dutch experience. Ethn Health. 2009;14(3):255–69. doi: 10.1080/13557850802509206 19052941 10.1080/13557850802509206

[12] CamNtech Ltd. The Actiheart User Manual 4.0.109. 2013.

[13] Brage S, Brage N, Franks PW, Ekelund U, Wareham NJ. Reliability and validity of the combined heart rate and movement sensor Actiheart. Eur J Clin Nutr. 2005;59(4):561–70. doi: 10.1038/sj.ejcn.1602118 15714212 10.1038/sj.ejcn.1602118

[14] Collings PJ, Wijndaele K, Corder K, Westgate K, Ridgway CL, Sharp SJ, et al Prospective associations between sedentary time, sleep duration and adiposity in adolescents. Sleep Med. 2015;16(6):717–22. doi: 10.1016/j.sleep.2015.02.532 25959093 10.1016/j.sleep.2015.02.532 PMC4465960

[15] World Health Organization. Global Recommendations on Physical Activity for Health. 2010. 26180873

[16] World Health Organization. Obesity: preventing and managing the global epidemic. 2000. 11234459

[17] Rumbaut RG. Ages, Life Stages, and Generational Cohorts: Decomposing the Immigrant First and Second Generations in the United States. International Migration Review. 2004;38(3):1160–205.

[18] Brug J, van Stralen MM, Chinapaw MJ, De Bourdeaudhuij I, Lien N, Bere E, et al Differences in weight status and energy-balance related behaviours according to ethnic background among adolescents in seven countries in Europe: the ENERGY-project. Pediatr Obes. 2012;7(5):399–411. doi: 10.1111/j.2047-6310.2012.00067.x 22730265 10.1111/j.2047-6310.2012.00067.x

[19] van Rossem L, Vogel I, Moll HA, Jaddoe VW, Hofman A, Mackenbach JP, et al An observational study on socio-economic and ethnic differences in indicators of sedentary behavior and physical activity in preschool children. Prev Med. 2012;54(1):55–60. doi: 10.1016/j.ypmed.2011.10.016 22064316 10.1016/j.ypmed.2011.10.016

[20] Hornby-Turner YC, Hampshire KR, Pollars TM. A comparison of physical activity and sedentary behaviour in 9–11 year old British Pakistani and White British girls: a mixed methods study. Int J Behav Nutr Phys Act. 2014;11:74 doi: 10.1186/1479-5868-11-74 24912651 10.1186/1479-5868-11-74 PMC4059029

[21] Brodersen NH, Steptoe A, Boniface DR, Wardle J. Trends in physical activity and sedentary behaviour in adolescence: ethnic and socioeconomic differences. Br J Sports Med. 2007;41(3):140–4. doi: 10.1136/bjsm.2006.031138 17178773 10.1136/bjsm.2006.031138 PMC2465219

[22] Williams ED, Nazroo JY, Kooner JS, Steptoe A. Subgroup differences in psychosocial factors relating to coronary heart disease in the UK South Asian population. J Psychosom Res. 2010;69(4):379–87. doi: 10.1016/j.jpsychores.2010.03.015 20846539 10.1016/j.jpsychores.2010.03.015 PMC2946562

[23] Gilbert PA, Khokhar S. Changing dietary habits of ethnic groups in Europe and implications for health. Nutr Rev. 2008;66:203–15. doi: 10.1111/j.1753-4887.2008.00025.x 18366534 10.1111/j.1753-4887.2008.00025.x

[24] Nicolaou M, Doak CM, van Dam RM, Brug J, Stronks K, Seidell JC. Cultural and social influences on food consumption in dutch residents of Turkish and moroccan origin: a qualitative study. J Nutr Educ Behav. 2009;41(4):232–41. doi: 10.1016/j.jneb.2008.05.011 19508928 10.1016/j.jneb.2008.05.011

[25] Dekker LH, Nicolaou M, van Dam RM, de Vries JH, de Boer EJ, Brants HA, et al Socio-economic status and ethnicity are independently associated with dietary patterns: the HELIUS-Dietary Patterns study. Food Nutr Res. 2015;59:26317 doi: 10.3402/fnr.v59.26317 26041009 10.3402/fnr.v59.26317 PMC4454783

[26] de Munter JSL, van Valkengoed IGM, Agyemang C, Kunst AE, Stronks K. Large ethnic variations in recommended physical activity according to activity domains in Amsterdam, the Netherlands. Int J Behav Nutr Phys Act. 2010;7:85 doi: 10.1186/1479-5868-7-85 21114828 10.1186/1479-5868-7-85 PMC3004814

[27] Matthews CE, George SM, Moore SC, Bowles HR, Blair A, Park Y, et al Amount of time spent in sedentary behaviors and cause-specific mortality in US adults. Am J Clin Nutr. 2012;95(2):437–45. doi: 10.3945/ajcn.111.019620 22218159 10.3945/ajcn.111.019620 PMC3260070

[28] O'Donoghue G, Perchoux C, Mensah K, Lakerveld J, van der Ploeg H, Bernaards C, et al A systematic review of correlates of sedentary behaviour in adults aged 18–65 years: a socio-ecological approach. BMC Public Health. 2016;16(1):163 doi: 10.1186/s12889-016-2841-3 26887323 10.1186/s12889-016-2841-3 PMC4756464

[29] Katzmarzyk PT. Standing and mortality in a prospective cohort of Canadian adults. Med Sci Sports Exerc. 2014;46(5):940–6. doi: 10.1249/MSS.0000000000000198 24152707 10.1249/MSS.0000000000000198

[30] van der Ploeg HP, Chey T, Ding D, Chau JY, Stamatakis E, Bauman AE. Standing time and all-cause mortality in a large cohort of Australian adults. Prev Med. 2014;69:187–91. doi: 10.1016/j.ypmed.2014.10.004 25456805 10.1016/j.ypmed.2014.10.004

[31] PALtechnologies. Products 2015. http://www.paltechnologies.com/products/.

